# Review of Systemic Antibiotic Treatments in Children with Rhinosinusitis

**DOI:** 10.3390/jcm8081162

**Published:** 2019-08-03

**Authors:** Sara Torretta, Lorenzo Drago, Paola Marchisio, Lorenzo Gaini, Claudio Guastella, Antonio Moffa, Vittorio Rinaldi, Manuel Casale, Lorenzo Pignataro

**Affiliations:** 1 Fondazione IRCCS Ca’ Granda Ospedale Maggiore Policlinico, Milan 20100, Italy; 2 Department of Clinical Sciences and Community Health, University of Milan, Milan 20100, Italy; 3 Department of Biomedical Sciences for Health, University of Milan, Milan 20100, Italy; 4 Department of Pathophysiology and Transplantation, University of Milan, Milan 20100, Italy; 5 Unit of Otolaryngology, University of Foggia, Foggia 71121, Italy; 6 Unit of Otolaryngology, UOS ORL TI, Campus Bio-medico University, Rome 00128, Italy

**Keywords:** rhinosinusitis, children, antibiotics, infection

## Abstract

Antibiotic treatment in paediatric rhinosinusitis is still a matter of debate, as the current guidelines have been drafted mainly based on clinical studies published before 2013. Recent modifications in the epidemiological basis of the disease might mean that current treatments are not completely adequate considering the evolving microbiological profile of the disease. The present paper reviews the role of systemic antibiotics in children with acute (ARS), chronic (CRS), recurrent (RARS), and complicated acute (CoARS) rhinosinusitis. A total of 14 studies (including 3 prospective non-randomised studies, 8 retrospective studies, and 3 prospective randomised studies) of the 115 initially identified papers were included in this review, corresponding to 13,425 patients. Five papers dealt with ARS, four papers with RARS or CRS, and five papers with CoARS; the remaining papers included patients with either ARS or CRS. Data about the effectiveness of antibiotic treatment in children with ARC, CRS, and CoARS is scarce, as only three randomised controlled trials have been published in the last decade, with contrasting results. There is an urgent need for dedicated controlled trials not only to test the actual clinical benefits deriving from the routine use of systemic antibiotics in different categories of patients but also to compare the effectiveness of various therapeutic protocols in terms of the type of antibacterial molecules and the duration of treatment.

## 1. Introduction

Rhinosinusitis (RS) is a frequent condition in the paediatric population [1]. Acute RS (ARS) is generally secondary to bacterial superinfection after an acute upper respiratory tract infection (URTI) [1]. It is a relatively common condition, as it has been reported to develop in approximately 6.5% of children with upper respiratory tract infection [2], accounting for more than 20% of antibiotic prescriptions in children [1].

Medical protocols in children with ARS mainly include amoxicillin alone or in combination with clavulanic acid [3], but given that ARS generally follows a viral URTI, with signs and symptoms frequently overlapping, antibiotic treatment is probably overused, with possible side effects and concerns for the development of bacterial resistance and increasing health costs.

Chronic rhinosinusitis (CRS) is defined by the persistence of symptoms suggestive of nasal and sinusal inflammation lasting more than 12 weeks [4]. In some children with CRS, symptoms are not controlled despite adequate medical treatment based on guidelines. This subset of patients may be scheduled among those with the so-called paediatric severe chronic upper airway disease (P-SCUAD) [5].

CRS is a heterogeneous spectrum of disease, and different phenotypes and endotypes (i.e., biological subtypes defined by corresponding biomarkers and peculiar responsiveness to some medical treatments) may be identified [6]. Causative factors include allergic and non-allergic rhinitis, airway pollution, smoke exposure [7], bacterial biofilm [8,9], and anatomic anomalies impairing the sinonasal drainage. Underlying systemic diseases (such as cystic fibrosis, primary ciliary dyskinesia, immune defects) may also predispose to CRS [10,11]. The mainstream treatment of paediatric CRS consists of maximal medical therapy including 3 to 6 weeks of broad-spectrum systemic antibiotics with adjunctive therapies [12,13]. However, a recent survey evaluating the management of CRS in children in different paediatric healthcare providers [14] documented that the antibiotic protocols used are not completely homogeneous, with the most frequently used being amoxicillin (72%), amoxicillin-calvulanic acid (98%), cefdinir (73%), and azithromycin (15%), and that the length of treatment is generally 10 (70%) or 14 (17%) days. In the case of incomplete recovery after maximal medical therapy, surgery should be offered [15].

**Figure 1:**
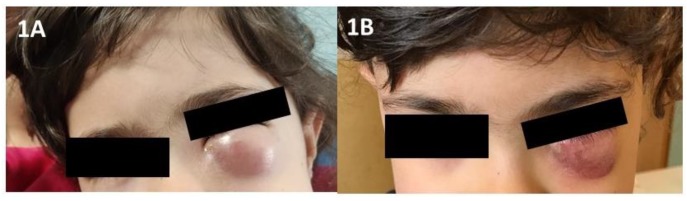
A 4-year-old girl with left periorbital cellulitis related to acute ethmoiditis before (**A**) and after (**B**) treatment with cefotaxime.

Complicated disease (CoARS) develops when the infection spreads to the nearby anatomic structures, including the orbits and the brain, possibly resulting in blindness and life-threatening sequelae [16]. Sinonasal-related orbital infections (SROIs) are the most frequent complications among children [1]: they typically follow an acute ethmoiditis and generally affect children younger than 5 years old [17]. SROIs comprise a large spectrum of clinical manifestations that can involve either the eyelids and adnexa anterior to the orbital septum or, more dangerously, the orbital structures placed behind it. Therefore, we can distinguish between pre-septal complications (i.e., periorbital cellulitis (POC) (Figure 1A,B) and post-septal complications that include orbital cellulitis (OC), sub-periosteal abscess (SPA), and orbital abscess (OA) [18]. Intra-cranial life-threatening complications are cerebral abscess, cavernous sinus thrombosis, and meningitis. Current evidence suggests that parenteral medical treatment should be considered first in patients with POC and OC [16]; scheduled protocols include clindamycin plus third-generation cephalosporin, vancomycin with or without meropenem, ampicillin-sulbactam, and third-generation cephalosporin plus metronidazole [19,20,21,22,23,24,25,26,27]. In the case of SPA, OA, or intracranial extension, urgent surgical drainage should be offered, too.

## 2. Microbiological Basis

Bacteriological analysis is highly suggested in order to adjust the antibiotic treatment. Polymicrobial or culture-negative specimens are not so rare; sterile samples are obtained in 25–30% of cases. In recent years, there has been an increase in the reports of fungal invasion accompanying rhinosinusitis. These forms are connected with several promoting factors such as diabetes, steroidotherapy, and immunosuppression [28].

The most common germs are *Streptococcus* species, anaerobes, and *Staphylococcus Aureus* [25,26,29]. In the medical literature, the aerobic species cited as being among the most prevalent include *S. aureus*, *H. influenza*, *S. pneumoniae*, *S. viridans,* and *M. catarrhalis*. The most frequently reported anaerobes include *Peptostreptococcus*, *Prevotella*, *Fusobacterium*, *Propionibacterium,* and *Bacteroides*. A recent classification [2] has elucidated the change in microbial population compared to the past and the microbial aetiological differences existing between acute and chronic rhinosinusitis in children. Recent publications have emphasised *S. milleri* as the most common pathogen in patients with complicated sinusitis [30,31,32].

Several studies have suggested a link between biofilm and rhinosinusitis. Yet, little is known about the pathophysiology and role of biofilm in the complicated forms of rhinosinusitis, and no studies on the factors that determine the formation and persistence of biofilms on the sinonasal mucosa and connected sites have clearly demonstrated its role.

Biofilms are morphologically characterised as three-dimensional complexes of bacteria enclosed in a self-produced extra-cellular matrix of polysaccharides, nucleic acids, proteins, and extra-cellular DNA. Most of the biofilm mass consists of water; the rest is made up of extra-cellular matrix and bacterial cells [33].

It is likely that biofilms occur in many cases of rhinosinusitis in complicated forms, but a methodology for processing clinical samples is currently lacking. Several groups have suggested using confocal scanning laser microscopy and fluorescent in situ hybridisation probes as the ‘gold standard’ for biofilm imaging. However, this should be combined with other microbiological tests, such as the traditional culture techniques used to identify and quantify pathogens [34].

Given the difficulty of studying biofilms in a viable tissue culture or even in animals, only a few studies have successfully evaluated their interaction with their host. Using human broncho-epithelial cells, Starner et al. showed that *H. influenzae* biofilms stimulate an inflammatory response by increasing the levels of NF-kB, IL-8, TNF-α, and the macrophage inflammatory protein MIP-3α.

In order to further investigate the behaviour of sinonasal mucosa biofilms, some studies have used animal models. Perloff and Palmer found that maxillary sinusitis induced by *P. aeruginosa* in rabbits was associated with biofilm formation on the sinus mucosa at different endpoints up to the 20th day of infection, and substantially promoted sinus ostium occlusion [35,36].

Many scientists are more convinced that the main negative result in rhinosinusitis patients with biofilm-endowed bacteria is the high degree of insusceptibility to several antibiotics as well as to the host immune mechanisms [37].

Chiu et al. [38] developed an animal biofilm sinusitis model in which an in-dwelling irrigation catheter is used to screen the anti-biofilm activity of antibiotics and/or surfactants. This model may be useful in the future development of topical drugs and for assessing the efficacy and safety of anti-biofilm agents such as anti-quorum-sensing drugs [39].

Familiarity with the microbiology of rhinosinusitis and its complications is of great importance for selecting empirical antimicrobial therapy. The selection of the appropriate antimicrobial agents is generally a first-line mandatory approach to obtain optimal efficacy (knowing microbes for choosing the best antibiotic). The main pathogenic factor that concurs with the negative outcome of the disease is certainly the biofilm, which necessarily requires a different diagnostic and therapeutic approach. The therapeutic intervention should act in two possible ways: (A) by inhibiting biofilm formation, or (B) by eradicating the biofilm already formed. Further studies are necessary in order to investigate the mechanisms underlying biofilm formation and the related cellular and humoral defence responses, as well as those factors driving the host-biofilm interactions. This will require the development of new and more feasible methods for detecting biofilm that should eventually lead to new therapeutic perspectives.

## 3. Aim of Study and Methods

Antibiotic treatment in paediatric RS is still a matter of debate because, despite the availability of guidelines on management strategies, the current guidelines mainly refer to clinical studies published before 2013. The microbiological profile of RS has evolved significantly due to the introduction of the pneumococcal conjugate vaccine (PCV) and changes in antibiotic susceptibility, which makes previously established guidelines inadequate for managing the current clinical manifestations.

The present paper discusses the role of systemic antibiotics in children with acute (ARS), chronic (CRS), recurrent (RARS), and complicated acute (CoARS) rhinosinusitis. Relevant original papers concerning the use of systemic antibiotic treatments in children with RS were selected by Sara Torretta after a MEDLINE search (accessed via PubMed) based on the terms “antibiotic and rhinosinusitis and children” on 21 March 2019. The articles searched aimed to evaluate the effectiveness and safety of systemic antibiotic treatments in children with ARC, RARS/CRS, and CoARS, as attested by the reported success rate in achieving clinical or symptomatic improvement, and related failure and side effects rate when available.

Consideration was only given to original clinical studies on human subjects published in the English language in peer-reviewed journals from March 2009. Animal studies, reviews, and case series including children with systemic disease (cystic fibrosis, primary ciliary dyskinesia, immunological defects) or adult patients were excluded.

The reference lists of identified articles were subsequently reviewed to ensure that all of the selected papers were truly relevant and to identify any articles that had possibly been overlooked.

## 4. Results

**Figure 2:**
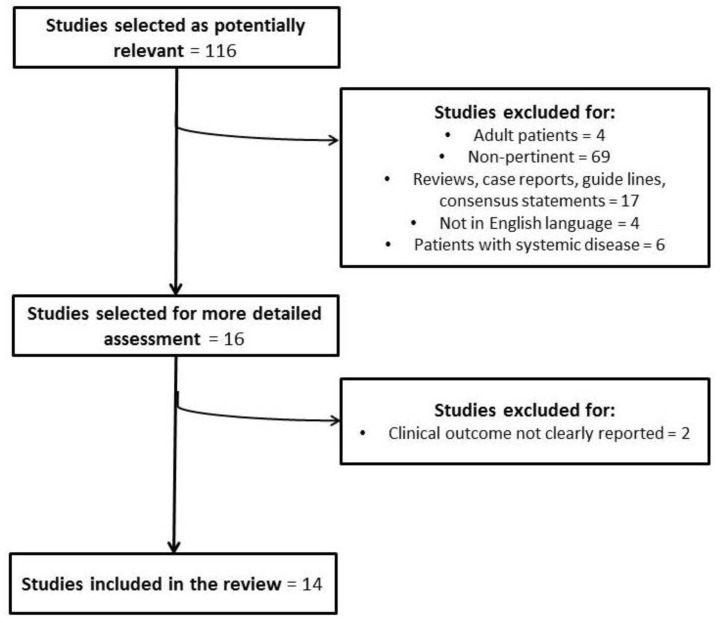
Flow-chart of article selection.

A total of 14 papers (including 3 prospective non-randomised studies, 8 retrospective studies, and 3 prospective randomised studies) of the 115 initially identified papers were included in this review, corresponding to 13,425 patients (Figure 2) [2,40,41,42,43,44,45,46,47,48,49,50,51]. Five papers dealt with ARS, four papers with RARS or CRS, and five papers with CoARS (in one study, patients with either ARS or CRS were included) [44].

•  Acute Rhinosinusitis

Among the five identified papers on systemic antibiotic treatments in children with ARS (and corresponding to 13,000 patients in total), we found one prospective non-randomised, two prospective randomised, and two retrospective studies. The studies were not homogeneous in terms of interventions or clinical outcomes. Table 1 shows the main characteristics of the selected case series and related results.

The study published by de Moor et al. [40] was a large retrospective assessment using the General Practice Research database from the United Kingdom to evaluate the comparative effectiveness of nasal steroid spray versus systemic antibiotics versus combined treatment in both paediatric and adult patients (12,679). It was found that the use of nasal steroid spray (alone or associated with antibiotic) was more effective in reducing rhinosinusitis-related medical encounters and decreasing the use of related prescriptions as compared to antibiotics alone.

The randomised, double-blind placebo-controlled trial by Wald et al. [2] evaluated the effectiveness of antibiotic therapy with amoxicillin (90 mg/kg/day) plus potassium clavulanate (6.4 mg/kg/day) in two doses compared to placebo in 56 children with ARS. They found a significantly increased cure rate and reduced failure rate in the study group compared to controls; on the other hand, side effects were more frequent in children receiving antibiotics.

More recently, a prospective, randomised, blind-controlled trial by Ragab et al. [41] compared the effectiveness of amoxicillin (100 mg/kg/day) plus 0.9% nasal saline irrigations against placebo plus 0.9% nasal saline irrigations on 62 patients. They found that the clinical cure (defined as complete recovery of all symptoms and signs of infection) was slightly, although not significantly, increased in the study group compared to controls. No difference between the groups was detected in terms of subjective improvement (attested by the nasal symptoms score and the standardised Paediatric Rhinoconjunctivitis Quality of Life Questionnaire), but the side effects rate increased in the control group compared to the placebo group.

•  Recurrent Acute Rhinosinusitis and Chronic Rhinosinusitis

Among the four identified papers (and corresponding to 263 patients in total), we found one retrospective, one prospective randomised, and two prospective non-randomised trials. The studies were not homogeneous in terms of interventions or clinical outcomes. Table 2 shows the main characteristics of the selected case series and related results.

Two papers [42,43] assessing the reduction in sinonasal infectious exacerbations in children with RARS documented a positive effect of the treatment protocol in most cases. In particular, Veskitkul et al. [43] published a randomised double-blind placebo-controlled trial in 60 children receiving azithromycin 5 mg/kg/day for 3 days a week for 12 months or placebo. They reported a significantly reduced number of acute infectious episodes in the study group compared to the placebo group, and a significant improvement in terms of symptom and adjunctive medication scores compared to baseline in the treatment group but not in the control group.

Two papers [44,45] on the effectiveness of systemic antibiotics using different therapeutic protocols in children with CRS found a symptomatic improvement in most cases. Shin et al. [45] attributed clinical failure to the presence of increased serum eosinophils or eosinophilic cationic protein levels. No comparative evaluations of different therapeutic protocols were performed. 

•  Complicated Acute Rhinosinusitis

All five of the identified papers (corresponding to 162 patients in total) were retrospective studies. The studies were not homogeneous in terms of interventions or clinical outcomes. Table 3 shows the main characteristics of the selected case series and related results.

Two papers [46,47] reported clinical outcomes in children with sinonasal-related subperiosteal orbital abscesses taking systemic antibiotics (with different therapeutic protocols) with or without complementary surgery. A positive response to conservative treatment in children with a small abscess (i.e., volume < 0.5 mL) was documented by Gavriel et al. [46]. On the contrary, Ketenci et al. [47] reported the development of recurrences in more than 8% of cases, and the occurrence of life-threatening events including unilateral blindness (5.5%), intracranial abscess formation, and death (2.8%) despite systemic antibiotic and surgical treatment.

Schupper et al. [48] retrospectively assessed clinical outcomes in children with sinonasal-related intracranial complications and documented recurrences with abscess reaccumulation in approximately 37% of patients receiving vancomycin, meropenem, or ceftriaxone with surgery.

## 5. Discussion

Systemic antibiotic therapy in paediatric ARS is currently based on the guidelines of the Infectious Disease Society of America and American Academy of Paediatric, respectively published in 2012 and 2013 [52,53]. The former suggests amoxicillin-clavulanate (rather that amoxicillin alone) as an empiric antibiotic treatment in children with ARS (at the dosage of 90 mg/kg/day in two doses in children with severe disease or with a risk factor for invasive penicillin-nonsusceptible *S. pneumoniaea*) for 10–14 days [52]; the latter also includes high-dose amoxicillin as a first-line treatment for 10–28 days [53] (Table 4). More recently, Wald et al. [54] proposed a regular dose (45 mg/kg/day in two doses) of amoxicillin-clavulanate as the preferred treatment for paediatric ARS; this approach results from the observation that, since the licensure of 7-valent PCV (PCV7), and then 10-valent PCV (PCV10) and 13-valent PCV (PCV13), the rate of nasopharyngeal colonisation with vaccine strains of *S. pneumoniaea* has declined, and although new serotypes have emerged, the prevalence of penicillin-resistant *S. pneumoniae* is low, so the use of high-dose amoxicillin is not mandatory. On the other hand, the subsequent increase in *H. influenza* and *M. catarrhalis* (able to produce beta-lactamase) as causative germs requires association with clavulanic acid in order to enhance beta-lactamase coverage.

In 2014, a panel composed of nine representative otolaryngologists produced a consensus statement about the diagnostic and therapeutic management of CRS [55]. They did not reach agreement about the appropriate antibiotic treatment including a minimum of 10 consecutive days, but they agreed on the superiority of a 20-day compared to a 10-day treatment regimen of systemic antibiotic treatment and on the fact that culture-directed antibiotic therapy may improve clinical outcomes in patients not responding to empirical treatment for CRS [55]. More recently, a review [56] about the role of antibiotic treatment in paediatric CRS identified three possible alternative oral treatment regimens effective against polymicrobial infections possibly substained by beta-lactamase-producing aerobic and anaerobic pathogens. They included amoxicillin-calvulanate as the first-line option (at the dosage of 45 mg/kg/day divided into two doses; the 90-mg/kg/day dose should be used in children from geographic areas with a high endemic rate of invasive penicillin-nonsusceptible *S. pneumoniae* and in those with severe infection, attending daycare, younger than 2 years, with immune defects, recent hospitalisation, or antibiotic assumption) or clindamycin (20–40 mg/kg/day divided into a dose every 6–8 h) in penicillin-allergic children. Refractory cases should be treated with metronidazole (30–50 mg/kg/day divided into three daily doses) plus one molecule that is active against aerobic and facultative bacteria (cefuroxime axetil, cefdinir, cefpodoxime proxetil, azithromycin, clarithromycin, trimethoprim-sulfamethoxazole) [56].

Our review documents the paucity of high-quality evidence deriving from papers published in the last decade about the antibiotic management of children with RS. In particular, no randomised controlled studies of good quality have been recently conducted to test the applicability of antibiotic protocols in children with CRS or CoARS. Only two randomised controlled trials on paediatric ARS were published in the last 10 years [2,41], both of them evaluating the effect of amoxicillin (with or without clavulanic acid 100 mg/kg/day) with or without complementary therapy, with contrasting results. In fact, the study by Wald et al. [2] favoured treatment, while a more recent study by Ragab et al. [41] found no significant difference in the cure rate between children receiving the antibiotic (plus nasal saline irrigations) and those receiving the placebo (plus nasal saline irrigations). A further study retrospectively comparing the cure rate in ARS children (with or without concomitant allergic rhinitis) undergoing different therapeutic protocols found better results in children receiving amoxicillin-clavulanic acid (with or without intranasal corticosteroids) compared to those receiving a placebo. As a whole, the reported recovery rates are greater in the antibiotic groups (50–94%) compared to placebo groups (14–71%), and side effects were more common in the former group (44–58% vs. 14–26%).

In addition, despite knowing about the involvement of bacterial biofilms in the pathogenesis of RS in children and that they may impair the effectiveness of traditional systemic antibiotic treatments [8,9], not one of the clinical studies reviewed here reported the diagnosis of biofilms. Accordingly, we suggest incorporating biofilm detection in patients with chronic or recurrent disease, either by the means of electron microscopy analysis on bioptic mucosal specimens taken during surgery in child candidates for adenoidectomy or endoscopic sinus surgery after the failure of maximal medical treatment, or by the means of a simpler spectrophotometric evaluation performed on endoscopically-collected middle meatal swabs. Moreover, we strongly advocate dedicated clinical studies aimed at evaluating the responsiveness of different medical protocols in patients with biofilm-related CRS or RARS.

## 6. Conclusions

Today, antibiotic treatment in paediatric RS is mainly based on guidelines published in 2012 and 2013, which might no longer be completely appropriate, given the supposed change in bacterial etiology after the introduction of the pneumococcal conjugate vaccine, promoting the emergence of non-vaccinal serotypes, and the absence of dedicated microbiological studies evaluating the role of new emerging pathogens and related resistance.

There are very few recent studies on the effectiveness of antibiotic treatment in children with ARC, CRS, and CoARS, as only three randomised controlled trials have been published in the last decade, with contrasting results.

There is an urgent need for dedicated controlled trials not only to test the actual clinical benefits of the routine use of systemic antibiotics in different categories of patients but also to compare the effectiveness of various therapeutic protocols in terms of the kind of antibacterial molecules and duration of treatment.

## Figures and Tables

**Table 1:** Results of the included studies on ARS.

Author, Year		Pts Category	No. of Pts; Age (Years)	Study Design	Interventions	Outcomes	Failures and Side Effects
Wald et al., 2009 [2]			56; 66 ± 30 months	Prospective randomised	Amoxicillin (90 mg/kg/day) with potassium clavulanate (6.4 mg/kg/day) in two doses (50%) vs. placebo	Children receiving antibiotics were more likely to be cured (50% vs. 14%; *p* = 0.010) and less likely to have treatment failure (14% vs. 68%; *p* < 0.001) compared to placebo	Adverse events * more frequently occurred in treatment compared to placebo group (44% vs. 14%; *p* = 0.014); * self-limiting diarrhoea
De Moor et al., 2012 [40]			12679; 12–75	Retrospective	Mometasone furoato nasal spray (MNS) vs. MNS plus antibiotic vs. antibiotic	MNS (alone or in combination with antibiotic) significantly reduced the risk of rhinosinusitis-related medical encounters compared to antibiotic alone	
Poachanukoon et al., 2012 [44]		ARS (103) and CRS (51)	154; 5.9 ± 3.3	Prospective non-randomised	Amoxicillin-clavulanic acid (54.5%), cefditoren pivoxil (33.8%) for 14.6 ± 3.8 days (ARS) or 22.3 ± 5.0 days (CRS); intravenous antibiotic in 5.8% of cases (77.8% with ARS)	Symptomatic improvement in ARS group after 14.6 days of treatment on average and in CRS group after 22.3 days of treatment on average; no one underwent surgery	
Wan et al., 2015 [49]		Allergic rhinitis in 50%	100; 15 ± 1.7	Retrospective	Amoxicillin-clavulanic acid vs. amoxicillin-clavulanic acid plus intranasal corticosteroid vs. placebo	Symptom improvement in 92% and 88% of patients receiving amoxicillin-clavulanic acid with and without allergic rhinitis, respectively, compared to symptom improvement in 84% and 96% of patients receiving amoxicillin-clavulanic acid plus intranasal corticosteroid with and without allergic rhinitis, respectively; symptomatic improvement in 30% of placebo group	
Ragab et al., 2015 [41]			62; 5.4 ± 2.3 and 4.6 ± 2.0	Prospective randomised	Amoxicillin 100 mg/kg/day, three times a day, plus nasal saline irrigations vs. placebo plus nasal saline irrigations	Clinical recovery in 83.9% and 71% of patients respectively belonging to study and control group (not significant); clinical improvement in 3% and 6% of patients respectively belonging to study and control group	Adverse events in 58.1% * and 25.8% of patients respectively belonging to study and control group (*p* = 0.005); * including diarrhoea (29.0%), abdominal pain (16.1%), and nausea (6.0%)

ARS = acute rhinosinusitis; CRS = chronic rhinosinusitis; Pts = patients.

**Table 2:** Results of the included studies on RARS or CRS.

Author, Year	Pts Category	No. of Pts; Age (Years)	Study Design	Interventions	Outcomes	Failures and Side Effects
Poachanukoon et al., 2012 [44]	ARS (103) and CRS (51)	154; 5.9 ± 3.3	Prospective non-randomised	Amoxicillin-clavulanic acid (54.5%), cefditoren pivoxil (33.8%) for 14.6 ± 3.8 days (ARS) or 22.3 ± 5.0 days (CRS); intravenous antibiotic in 5.8% of cases (77.8% with ARS)	Symptomatic improvement in ARS group after 14.6 days of treatment on average and in CRS group after 22.3 days of treatment on average; no one underwent surgery	
Shin et al., 2015 [45]	CRS (62.1% non-respondents and 37.9% respondents)	58 with CRS; 5.8 ± 3.0 and 5.6 ± 2.7	Prospective non-randomised	Amoxicillin (90 mg/kg/day) with clavulanic acid (6.4 mg/kg/day), second-or third-generation cephalosporines for 12 weeks	Symptomatic, clinical, or radiological recovery in 62.1%	Lack of response to antibiotic treatment was significantly related to increased total eosinophilic count and serum eosinophilic cationic protein levels
Veskitkul et al., 2015 [42]	RARS (IgG subclass deficiency in 78.7%)	94; 7.7 ± 2.6	Retrospective	Oral antibiotic prophylaxis in 61.5% * (± adenotonsillectomy, allergen immunotherapy, gentamicin nasal irrigations, intravenous immunoglobulins); * amoxicillin or azithromycin	Symptomatic improvement in 80% of patients receiving antibiotic	
Veskitkul et al., 2017 [43]	RARS	60; 5–15	Prospective randomised	Azithromycin 5 mg/kg/day for 3 days a week for 12 months	Number of acute episodes significantly reduced in children receiving antibiotic compared to controls with a number needed to treat = 2; subjective improvement and reduced adjunctive medication in study but not control group	

RARS = recurrent acute rhinosinusitis; CRS = chronic rhinosinusitis; Pts = patients.

**Table 3:** Results of the included studies on complicated acute rhinosinusitis (CoARS).

Author, Year		Pts Category	No. of Pts; Age (Years)	Study Design		Interventions	Outcomes	Failures and Side Effects
Kristo et al., 2009 [50]		Orbital complications	20; 6.4 (8.0–12.4)	Retrospective		Parenteral antibiotic * (surgery in 10%); * cefuroxime (80.0%), clindamycin (10.0%), combined (10.0%)	Complete recovery in 100%	
Hurley et al., 2011 [51]		SPA	42; <9 years	Retrospective		Parenteral antibiotic: combined therapy with either amoxicillin-sulbactam or a third-generation cephalosporin, and either clindamycin or vancomycin for 2–8 days, followed by amoxicillin-clavulanate for 2–3 weeks	Complete recovery in 97.6%	One patient readmitted and underwent surgery
Gavriel et al., 2011 [46]		SPA	48; 4.0 ± 3.5	Retrospective		Parenteral antibiotic * (surgery in 47.6%); *amoxicillin-clavulanic acid (83.3%), ceftriaxone (10.0%), clindamycin plus metronidazole (8.3%), cefuroxime (2%)	Children with an abscess >0.5 mL should be candidates for surgery; conservative treatment can be considered only in those with smaller abscess without visual impairment	
Ketenci et al., 2013 [47]		SPA	36; 3–76 (47.2% < 10 years)	Retrospective		Ampicillin with or without metronidazole or clindamycin as first choice (ampicillin in 64.0%, multiple antibiotics* in 36.0%); * ampicillin-sulbactam, clindamycin, sulperazone, metronidazole; 3–9 days for medial, 6–10 days for superior, and 3–4 days for inferior SPA location	Visual recovery in 47.21%; partial visual recovery in 5.5%	Unilateral blindness in 5.5%. Early recurrences in 5.5% (second surgery); late recurrence in 2.8% (first surgery); intracranial abscess with fatal outcome in 2.8% (repeated surgery)
Schupper et al., 2018 [48]		Intracranial complications	16; 5–17	Retrospective		Parenteral antibiotic (vancomycin, meropenem, and ceftriaxone) with sinus surgery (plus craniotomy in 68.0%)		Abscess reaccumulation in 37.5%. Allergic reaction (12.0% to ceftriaxone, vamcomycin), meropenem-induced neutropenia (6.0%)

SPA = subperiosteal orbital abscess; Pts = patients.

**Table 4:** Indications for systemic antibiotic therapy in paediatric ARS based on guidelines.

Author, Year	Document	Recommendation
Chow et al., 2012 [52]	IDSA guideline	Amoxicillin-clavulanate. In patients with risk factors *, amoxicillin-clavulanate (90 mg/kg/day in two doses) or third-generation cephalosporin plus clindamycin for 10–14 days. In the case of non-type I hypersensitivity, third-generation cephalosporin plus clindamycin, doxycycline
Wald et al., 2013 [53]	AAP guidelines	Amoxicillin or amoxicillin-clavulanate in children with severe onset or worsening for 10–28 days. Intravenous or intramuscular ceftriaxone (50 mg/kg) if oral therapy is not possible. In the case of non-type I hypersensitivity cefdinir, cefuroxime; in the case of type-I hypersensitivity, cefdinir, cefuroxime or cefixime plus clindamycin
Wald and de Muri, 2018 [54]	Expert opinion	Amoxicillin-clavulanate (45 mg/kg/day) in two doses

IDSA = Infectious Diseases Society of America; AAP = American academy of paediatrics; * patients who received previous antibiotic, with severe infection, attendance to school, systemic disease, living in endemic area for invasive penicillin-nonsusceptible *S. pneumoniae*, younger than 2 years.
